# LONELINESS AND DEPRESSION AMONG SEXUAL AND GENDER MINORITY OLDER ADULTS WITH COGNITIVE IMPAIRMENT

**DOI:** 10.1093/geroni/igae098.3864

**Published:** 2024-12-31

**Authors:** Hyun-Jun Kim, Kilohana Haitsuka, Hailey Jung, Karen Fredriksen-Goldsen

**Affiliations:** University of Washington, Seattle, Washington, United States; University of Washington, Seattle, Washington, United States; University of Washington, Seattle, Washington, United States; University of Washington, University of WA, Goldsen institute, Washington, United States

## Abstract

Sexual and gender minority (SGM) older adults are at elevated risks of cognitive impairment (CI). High rates of loneliness and depression are particularly concerning for SGM older adults living with CI as they are linked to decreased physical health, lower life satisfaction, and accelerated cognitive decline. Yet little is known about the risk and protective factors of loneliness and depression for SGM older adults with CI. This study, utilizing the Health Equity Promotion Model, analyzes the Aging with Pride: National Health, Aging, and Sexuality/Gender Study to examine socioeconomic status, adverse life course experiences, and psychological and social factors that predict depressive symptomatology and the mediating role of loneliness. Data were a sub-sample of 160 participants with CI assessed by the Montreal Cognitive Assessment. Approximately 50% reported feeling isolated from others, 33% were experiencing depression, 40% reported physical or sexual child abuse, 70% were single, and 25% had a network size of 3 or less. A bootstrapped mediation analysis using Mplus revealed that child abuse, day-to-day discrimination, perceived stress, not being partnered, reduced network size, and lack of social support and community engagement were indirectly associated with depressive symptomatology via loneliness. Those with adverse childhood experiences, ongoing distressing stigma, and social isolation are of major concern. Improving support networks may reduce the risk of loneliness and depression. These findings can be utilized to develop culturally tailored interventions to enhance the mental health and overall quality of life of this understudied and underserved population.

